# Data on enrichment of chitosan nanoparticles for intranasal delivery of oligonucleotides to the brain

**DOI:** 10.1016/j.dib.2019.105093

**Published:** 2020-01-03

**Authors:** Vasyl Sava, Oksana Fihurka, Anastasia Khvorova, Juan Sanchez-Ramos

**Affiliations:** a Department of Neurology, University of South Florida, Tampa, FL, USA; b RNA Therapeutics Institute, University of Massachusetts Medical School, Worcester, MA, USA

**Keywords:** Nanocarriers, Chitosan, siRNA, Polyelectrolyte complexation, Enrichment

## Abstract

Data on preparation and characterization of chitosan-based nanoparticles (NP) carrying small interfering RNA (siRNA) for non-invasive gene therapy is presented. Polyelectrolyte complexation method was carried out in diluted concentrations to obtain relatively small (less than 200 nm) NP. To provide substantial dose of siRNA within tolerable volume of intranasal administration the NP were subjected to enrichment process. Offered here NP fabrication does two steps process comprise provisional and enriched preparations? The differences between these preparations were analyzed with hydrodynamic size distribution and zeta potential measurements. The effect of siRNA lipophilicity on NP physical instability was also tested. Biological evaluation of nanoparticles is described in our published article [1].

**Table:** Specifications Table

Subject	Nanobiotechnology, gene therapy, non-invasive delivery to the brain
Specific subject area	Fabrication of chitosan nanoparticles with enrichment processing for intranasal delivery of oligonucleotides to the brain
Type of data	Experimental protocols, Tables, Figures
How data were acquired	Enrichment performed with Eppendorf Vacufuge centrifugal evaporator (Eppendorf, N.Y). The hydrodynamic size distribution and zeta potential were measured by dynamic light scattering (DLS) using Malvern Zetasizer Nano ZS90 (Westborough, MA). Index of instability was measured using an analytical photo centrifuge LUMiSizer (LUM GmbH, Berlin, GER).
Data format	Raw and analyzed
Parameters for data collection	Data obtained by varying siRNA concentration from 10.9 to 130.5 μM, for provisional nanoparticle preparations and by 1.5, 3, 6, 9 and 12-fold enrichment for enriched preparation. Concentration of chitosan was varied reciprocally to obtain molecular ratio Chitosan/siRNA of 0.31.
Description of data collection	Data were collected by measurement of physical stability depending on chemical structure and concentration of ingredients employed for fabrication of nanoparticles.
Data source location	Department of Neurology, University of South Florida, Tampa, Florida, USA
Data accessibility	With article and Supplemental Data
Related research article	V. Sava, O. Fihurka, A. Khvorova, J. Sanchez-Ramos. Enriched Chitosan Nanoparticles Loaded with siRNA are Effective in Lowering Huntington's Disease Gene Expression Following Intranasal Administration. Nanomedicine: Nanotechnology, Biology, and Medicine 24, (2020) 102119

**Table:** 

**Value of the Data** • The paper relates to the research of nanoparticle-based biomaterials for biomedical applications. • This dataset presents a protocol for synthesis and characterization of a new bio-nanoparticles, which could be used by researchers in the same area. • The data include a preliminary assessment of novel nanoparticles for silencing of HTT gene in brain

## 1 Data

Here, we present data obtained on enrichment and characterization of chitosan-based NP carrying siRNA of different lipophilicity [1].

**Fig. 1:**
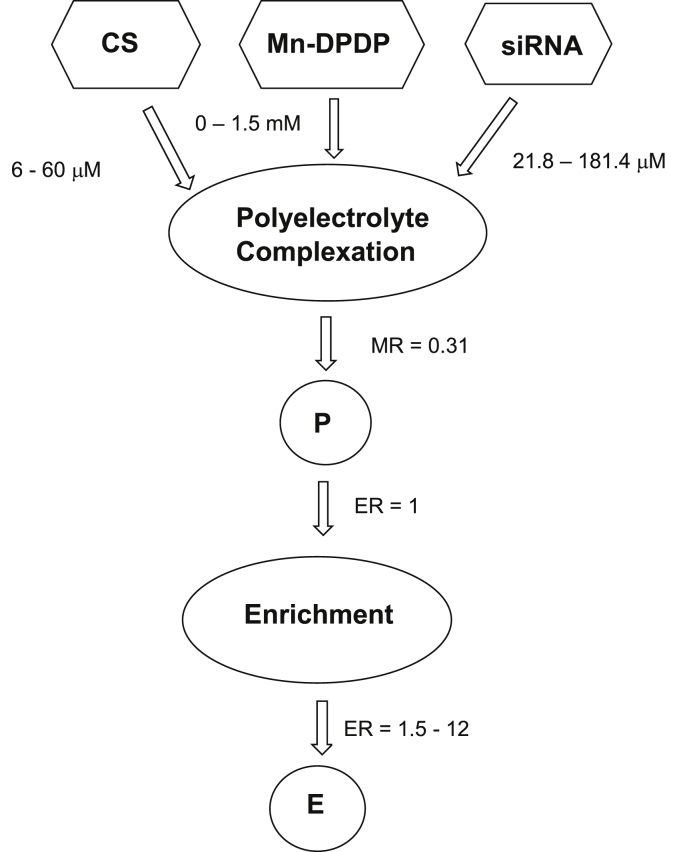
Flow chart of NP fabrication. Enrichment rate (ER) was calculated as quotient of P and E volumes.

Fig. 1 presents the flow chart of two-steps fabrication of NP comprising polyelectrolyte complexation of diluted ingredients including small interfering RNA (siRNA), Mn-Dipyridoxal diphosphate (Mn-DPDP) as crosslinker and chitosan (CS) resulted in formation of provisional nanoparticles (P) and enriched preparation (E).

Polyelectrolyte complexation reaction was performed with siRNA ranged from 10.9 to 90.7 μM of final concentration. The enriched nanoparticles E were fabricated from single P preparation obtained at the lowest concentration of siRNA (10.9 μM) by up to 12-fold enrichment processing that brought siRNA concentration to 130.5 μM.

**Fig. 2:**
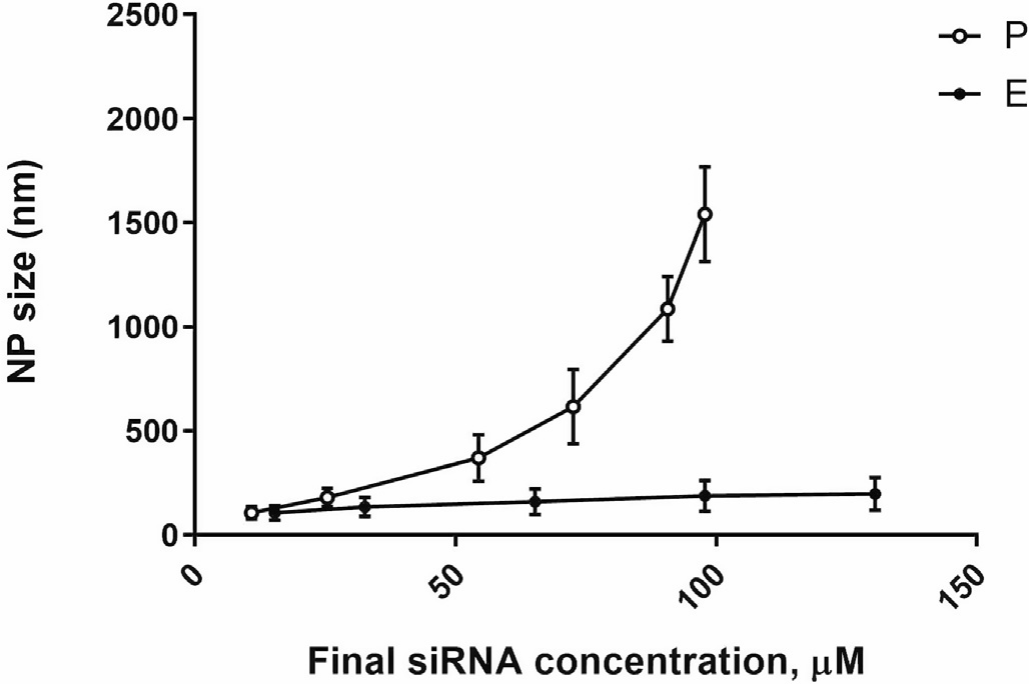
Changes of nanoparticle size for P (circles) and E (squares) preparations in relation with siRNA concentration. E nanoparticles were obtained from the lowest size of P preparation by enrichment procedure. Concentration of CS was as required to maintain molecular ratio of 0.31.

Carrying out reaction of polyelectrolyte complexation by increasing concentration of siRNA has caused exponential growth of NP size. Experimental results indicate (Fig. 2) that application of siRNA solutions at concentrations above 32 μM limits the ability to obtain an acceptable NP size (below 200 nm). Thus, reliable fabrication of chitosan-based nanoparticles is possible only in diluted concentrations of all components that raise volume of preparation. However, the tolerable volume is very limited for intranasal administration. To lower volume of NP preparation and to provide necessary dose of siRNA for intranasal administration P preparation was subjected to enrichment based on centrifugal evaporation of water. In the present study, the enrichment protocol has generated 1.5, 3, 6, 9 and 12-fold increased concentrations of siRNA in E preparation.

**Fig. 3:**
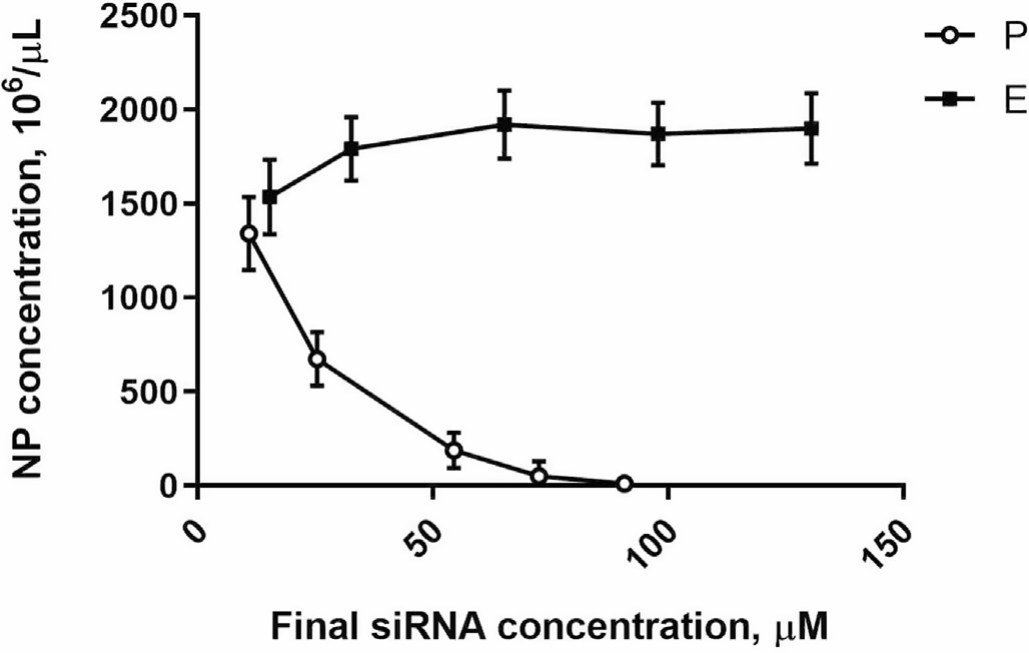
Changes of nanoparticle concentration in P (circles) and E (squares) preparations in relation with siRNA concentration.

The number of nanoparticles in given volume is getting low when concentration of ingredients for complexation reaction increases. Fig. 3 shows changes in NP numbers for both P and E preparations depending on siRNA content. Increasing the concentration of siRNA in reaction of complexation negatively affects the NP concentration due to exponentially growing size of NP (Fig. 2). Enrichment allows obtaining higher concentration of siRNA without substantial changing of the NP size.

Enrichment helps to increase siRNA dose delivered via nanoparticle intranasal administration. However, enrichment of nanoparticles may introduce some instability to preparation. Performed measurements did not discover any noticeable negative effect of enrichment on stability of nanoparticles. In case of using more hydrophilic siRNA (with no conjugated cholesterol), the enriched nanoparticles become even more stable as compared with provisional preparation. This is an important value of the proposed method of NP fabrication.

**Fig. 4:**
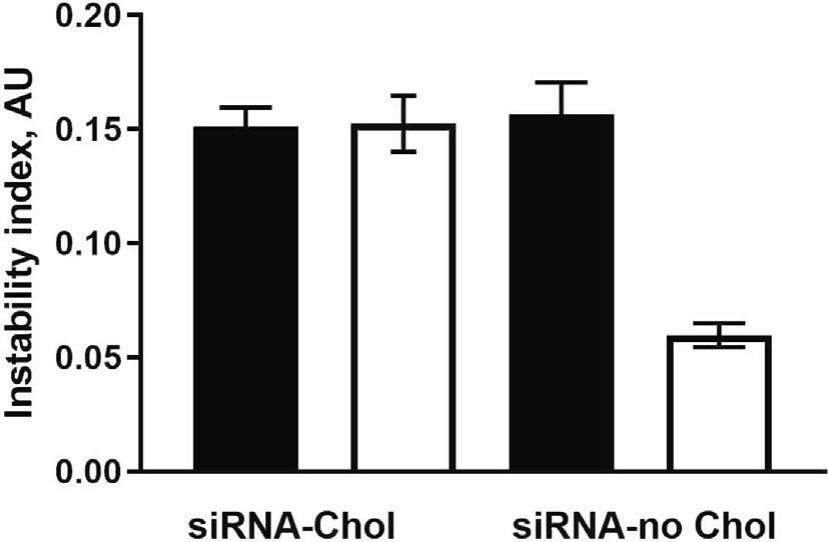
Physical instability of chitosan nanoparticles loaded with cholesterol contained siRNA (siRNA-Chol) and cholesterol-free (siRNA-no Chol). Closed and open bars represent P and E preparation, respectively.

Fig. 4 is depicted effect of enrichment on physical instability of NP in association with siRNA lipophilicity. The instability index [2] expressed in arbitrary units (AU) ranged from 0 (most stable) to 1 (most unstable).

## 2 Experimental design, materials, and methods

### 2.1 Polyelectrolyte complexation

For polyelectrolyte complexation the equal volumes of chitosan and siRNA solutions were blended under vigorous stirring at room temperature. Concentrations of CS varied from 6 to 60 μM. Concentrations of siRNA varied from 21.8 to 181.4 μM and were premixed with Mn-DPDP. Concentration of Mn-DPDP was from 0 to 1.5 mM depending on desired crosslinking density as previously described [3,4]. Molecular ratio (MR) between concentrations of chitosan and siRNA was kept optimal in all preparations. The value of optimal MR was established during preliminary investigation of most appropriate conditions for polyelectrolyte complexation leading to formation NP below 200 nm and with positive zeta-potential between 30 and 55 mV. It was established MR = 0.31 for present experiments.

### 2.2 Enrichment of nanoparticles for in vivo administration

The enrichment of NP was carried out with Eppendorf Vacufuge centrifugal evaporator (Eppendorf, N.Y) partially eliminating solvent from nanoparticle preparation. Evaporation temperature was set to 40 °C and centrifugation time was in range 2–4 hours at 1400 rpm. NP size of obtained E preparation was ranged from 90 to 200 nm with zeta potential between +42 and + 55 mV.

### 2.3 Materials

Chitosan (CS) of low molecular weight (60,000–120,000 Da with 85% deacetylation) was purchased from Sigma-Aldrich, MO. CS was dissolved in 0.5% acetic acid to obtain 60 μM stock solution that was passed through a syringe filter (pore size 0.2 μm, Millipore, USA) before dilution to working concentrations in ultrapure RNase free water.

Two different double stranded RNA oligonucleotides synthesized at the University of Massachusetts (UMASS) RNA Institute [5] were used for nanoparticle preparation. There were two types of siRNA designated for silencing HTT gene. One of them (cy3 HTT 10150- P2VP-Chol) contained cholesterol conjugated at the 3’ end of antisense strain. Another one (cy3 HTT 10150-P2VP) has 3'-end free. Stock concentration of siRNA was 196 μM prepared on RNase-free water.

Mn-DPDP was purchased from U.S. Pharmacopeial Convention (Rockville, MD). It was dissolved in ultrapure RNase free water to form 1.5. mM stock solution passed through a syringe filter (pore size 0.2 μm, Millipore, USA) before use.

## 3 Methods

### 3.1 Nanoparticle size and zeta potential

The hydrodynamic size distribution and zeta potential in NP preparations were determined at 25 °C by dynamic light scattering measurements with Malvern Zetasizer Nano ZS90 (Westborough, MA).

### 3.2 Physical stability of NP

Physical stability of the nanoparticles was determined on an analytical photo centrifuge LUMiSizer (LUM GmbH, Berlin, GER). Measurements were performed by using quantity of transmitted near infrared light (870 nm) as a function of time and position of sedimentation boundary in centrifuge cuvette. Testing parameters were as follows: cuvette volume – 200 μL; rotation speed – 4000 rpm; time interval – 30 s; number of measurements - 255; temperature - 25 °C. Special software SEP view 5.1 was used for the analysis.

## References

[1] Sava V., Fihurka O., Khvorova A., Sanchez-Ramos J. (2020). Chitosan nanoparticles loaded with siRNA are effective in lowering Huntington's disease gene expression following intranasal administration. Nanomed. Nanotechnol. Biol. Med..

[2] Wu L., Zhang J., Watanabe W. (2011 May 30). Physical and chemical stability of drug nanoparticles. Adv. Drug Deliv. Rev..

[3] Sanchez-Ramos J., Song S., Kong X., Foroutan P., Martinez G., Dominguez- Viqueria W. (2018). Chitosan-Mangafodipir nanoparticles designed for intranasal delivery of siRNA and DNA to brain. J. Drug Deliv. Sci. Technol..

[4] J. Sanchez-Ramos, V. Sava, S. Song, S. Mohapatra, S. Mohapatra, Divalent-metal coated nanoparticles for delivery of compositions into central nervous system by nasal insufflation, *Patent US 9,938,526 B2, issued: Apr. 2018, Washington, DC: US patent and Trademark Office*.

[5] Alterman J.F., Hall L.M., Coles A.H., Hassler M.R., Didiot M.C., Chase K., Abraham J., Sottosanti E., Johnson E., Sapp E., Osborn M.F., Difiglia M., Aronin N., Khvorova A. (2015. Dec 1). Hydrophobically modified siRNAs silence huntingtin mRNA in primary neurons and mouse brain. Mol. Ther. Nucleic Acids.

